# Supplementary material for: Inflammatory factors and risk of lung adenocarcinoma: a Mendelian randomization study mediated by blood metabolites
Source: Front Endocrinol (Lausanne). 2024 Aug 27;15:1446863. doi: 10.3389/fendo.2024.1446863 (PMC11384989; doi:10.3389/fendo.2024.1446863)
Supplement: Supplementary file 1 [file DataSheet1.zip › supplementary files/S2/GCST90200933/forest.pdf]

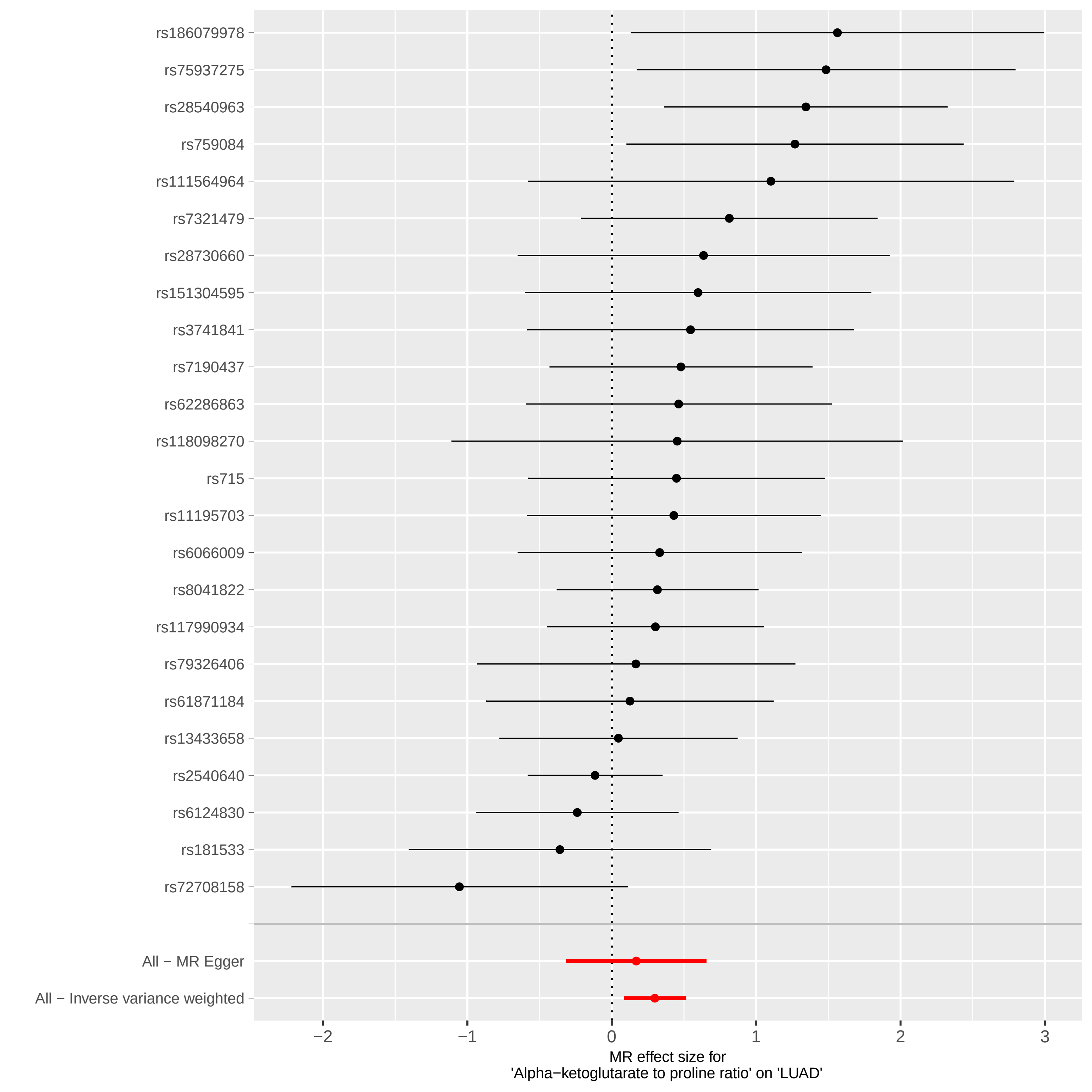
rs186079978
rs75937275
rs28540963
rs759084
rs111564964
rs7321479
rs28730660
rs151304595
rs3741841
rs7190437
rs62286863
rs118098270
rs715
rs11195703
rs6066009
rs8041822
rs117990934
rs79326406
rs61871184
rs13433658
rs2540640
rs6124830
rs181533
rs72708158
All − MR Egger
All − Inverse variance weighted
−2
−1
0
1
2
3
MR effect size for
'Alpha−ketoglutarate to proline ratio' on 'LUAD'
